# Sanguinarine Reduces Conjugative Transfer of *mcr-1*-Positive Plasmids in *Escherichia coli*

**DOI:** 10.3390/antibiotics15100973

**Published:** 2026-10-01

**Authors:** Xueyang Wang, Junfeng Zhang, Zhan Lin, Penghui Ru, Changsong Wang, Hewei Zhang

**Affiliations:** 1 College of Food and Drugs, Luoyang Polytechnic, Luoyang 471000, China; 202333008@lypt.edu.cn (X.W.); lzhan0410@163.com (Z.L.); penghui_ru@163.com (P.R.); 2 The 989th Hospital of the Joint Logistics Support Force of the Chinese People’s Liberation Army, Luoyang 471031, China; 3 College of Animal Science and Technology, Henan University of Science and Technology, Luoyang 471000, China; 9906470@haust.edu.cn

**Keywords:** sanguinarine, plasmid, conjugation, *mcr-1*, colistin, horizontal gene transfer, energy metabolism

## Abstract

Background/Objectives: IncI2 and IncX4 plasmids are important vehicles for dissemination of the mobile colistin resistance gene *mcr-1*. We evaluated whether sanguinarine (SA) reduces conjugative transfer of these two *mcr-1*-carrying plasmid types and examined biological changes accompanying the transfer phenotype. Methods: Conjugation was measured in *Escherichia coli* carrying pIncI2-*mcr-1* or pIncX4-*mcr-1* after exposure to 2–8 µg/mL SA. Growth-related measurements, intracellular ATP signal, glycogen content, enzyme activities, targeted energy metabolomics, RNA sequencing, reactive oxygen species (ROS), and membrane permeability were assessed, with biological profiling focused on the IncI2 system. pIncI2-*mcr-1* transfer was also examined in a mouse-gut model. Results: SA caused no detectable reduction in the reported OD_600_-based growth curves or viable donor/recipient counts under the tested conditions. At 8 µg/mL, SA reduced IncI2-*mcr-1* and IncX4-*mcr-1* conjugation frequency by approximately 176-fold and 117-fold, respectively. In the IncI2 system, lower luciferase-based ATP signals, glycogen accumulation, altered GAPDH and G6PDH activities, and decreased abundance of transfer-related transcripts accompanied the reduction in transfer. Targeted LC–MS/MS independently confirmed a dose-associated decrease in ATP and identified lower pools of several glycolysis-associated metabolites. In mice, the mean IncI2 transfer-frequency estimate was 8.41 × 10^−3^ in controls and 2.49 × 10^−3^ with SA (*n* = 10 per group; *p* < 0.0001). Conclusions: SA reduced transfer of the two tested *mcr-1*-carrying plasmids under the experimental conditions. The metabolic and transcriptional observations support an energy-associated explanatory model for the IncI2 system and highlight SA’s potential as a conjugation inhibitor to curb the spread of colistin resistance.

## 1. Introduction

Antimicrobial resistance (AMR) is a major global health burden, and bacterial AMR was estimated to be associated with 4.71 million deaths in 2021, including 1.14 million deaths directly attributable to resistance [1,2]. Resistance determinants can spread vertically within lineages and horizontally between bacteria, often through mobile genetic elements such as plasmids, transposons, and integrons [3]. Conjugative plasmids are particularly important contributors to antimicrobial resistance because they facilitate the horizontal spread of antibiotic resistance genes within and between bacterial species. They encode the machinery required to establish donor–recipient contact, process plasmid DNA, and transfer it through a type IV secretion system [4]. Antibiotic and non-antibiotic exposures can modify this process, sometimes increasing the transfer of antibiotic resistance genes (ARGs) at concentrations that do not measurably inhibit bacterial growth [5,6,7]. These observations have motivated strategies that directly target resistance dissemination without depending on conventional growth-inhibitory or bactericidal activity [8].

Colistin is used against severe infections caused by multidrug-resistant Gram-negative bacteria, but its utility is threatened by mobile colistin resistance genes [9]. Since the initial report of plasmid-mediated *mcr-1*, the gene has been detected in *Escherichia coli* and other Enterobacterales from human, animal, food, and environmental sources [10,11]. IncI2 and IncX4 plasmids are prominent *mcr-1* carriers, although their distribution, fitness, and transfer behavior vary among hosts and epidemiological settings [11,12,13]. Restrictions on colistin use have reduced some reservoirs, but *mcr-1*-carrying plasmids persist, and sub-inhibitory antimicrobial exposure can increase their transfer [12,14]. Compounds that reduce conjugation may therefore complement stewardship and surveillance, provided that their activity and biological boundaries are defined in the relevant plasmid–host systems.

Several small molecules have already been evaluated as conjugation modulators. Antiviral agents and melatonin reduce transfer in selected resistance-plasmid systems [15,16]. Dihydroartemisinin and chelerythrine reduce transfer of *mcr-1*-carrying plasmids and are accompanied by changes in bacterial energy-associated measurements [17,18]. Short-chain fatty acids inhibit conjugation in vitro and in chicken tissue explants, and acetylshikonin has been reported to inhibit several resistance plasmids through membrane- and energy-associated effects [19,20]. Thus, energy availability is an established theme in conjugation-inhibitor research rather than a mechanism unique to any one compound. The magnitude and direction of transfer effects can also depend on the plasmid, host, regulatory system, and experimental environment [7].

Sanguinarine (SA) is a benzylisoquinoline alkaloid and an active component of *Macleaya cordata* extracts. SA and related extracts have been studied for antimicrobial activity and for effects on intestinal physiology and microbiota in animals [21,22]. Repeated exposure can also select *E. coli* with increased SA resistance mediated by AcrB efflux, emphasizing that its biological effects and limitations require direct evaluation [23]. Whether SA alters conjugative transfer of the epidemiologically important IncI2- and IncX4-type *mcr-1* plasmids has not been defined.

In this study, we demonstrate that SA markedly inhibits the conjugative transfer of *mcr-1*-carrying IncI2 and IncX4 plasmids both in vitro and in a murine intestinal model. By combining measurements of intracellular ATP and glycogen, activities of key metabolic enzymes, and transcriptomic profiling of plasmid-encoded transfer elements, we show that SA perturbs central carbon metabolism and down-regulates genes essential for conjugation. These findings reveal a previously unrecognized function of SA as a conjugation inhibitor and suggest that repurposing a widely used feed additive could offer a feasible One Health strategy to curb the dissemination of *mcr-1* and prolong the clinical lifespan of colistin.

## 2. Results

### 2.1. SA Reduced Conjugative Transfer of Plasmid Carrying mcr-1

**Figure 1:**
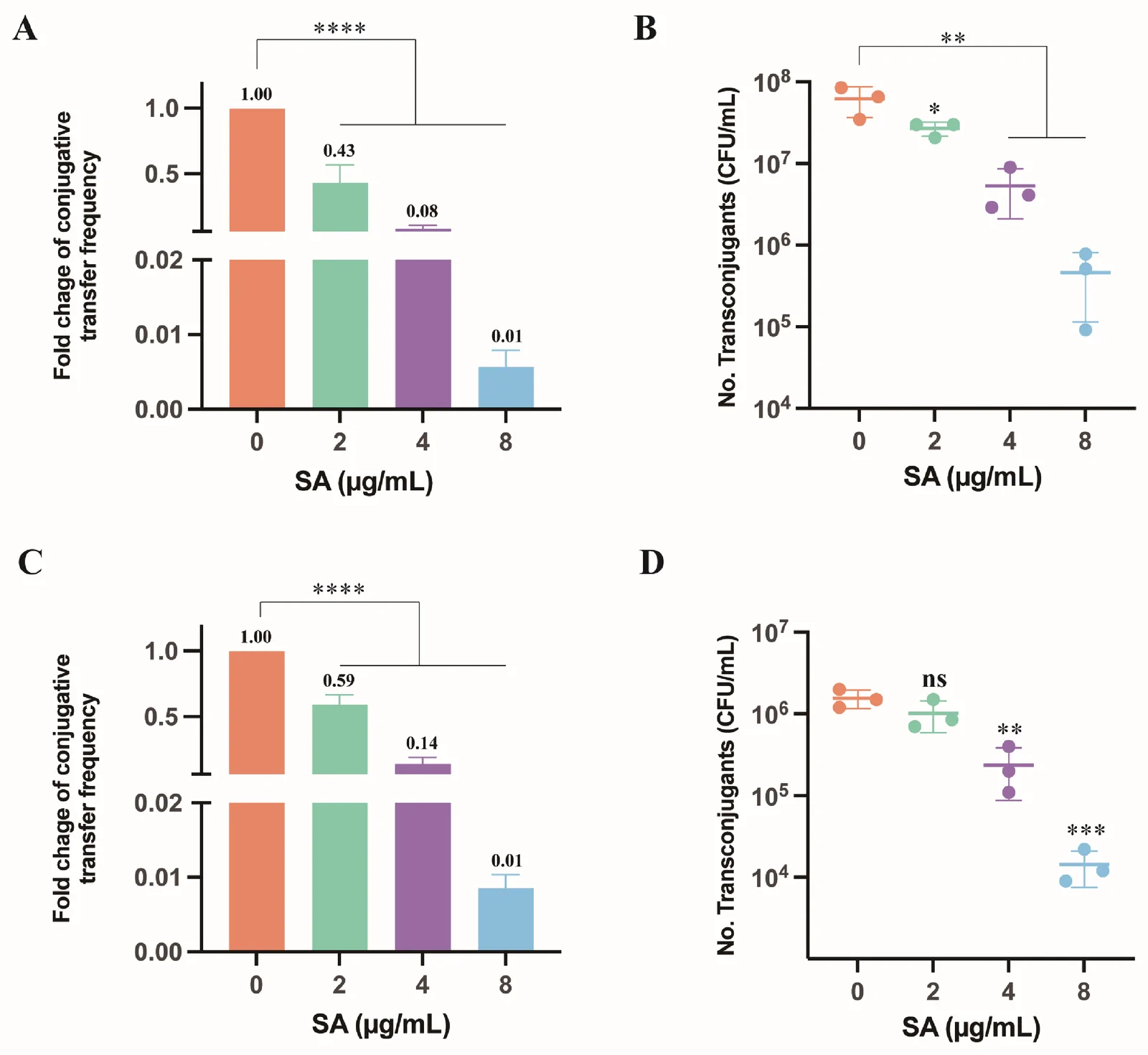
SA effects on conjugative transfer of the pIncI2-*mcr-1* and pIncX4-*mcr-1*. (**A**) Fold change in conjugative transfer frequency on the pIncI2-*mcr-1*. (**B**) The numbers of transconjugants of the pIncI2-*mcr-1*. (**C**) Fold change in conjugative transfer frequency on the pIncX4-*mcr-1*. (**D**) The numbers of transconjugants of the pIncX4-*mcr-1*. Transconjugant counts were determined by culture-based enumeration on selective agar. Data are mean ± SD from three biological replicates. One-way ANOVA with Dunnett’s multiple-comparisons test was used against the untreated control. Significance thresholds were defined as follows: ns, not significant, * *p* < 0.05, ** *p* < 0.01, *** *p* < 0.001, and **** *p* < 0.0001.

To eliminate the influence of SA on the growth of donor and recipient bacteria, a growth curve analysis was conducted on tested strains exposed to SA at various concentrations. The results of this analysis demonstrated that 2–8 μg/mL of SA has no significant bactericidal effect on the growth of donor and recipient bacteria (Figures S1 and S2). For the pIncI2-*mcr-1*, the control yielded 6.19 × 10^8^ transconjugants CFU/mL and a conjugation frequency of 1.65 × 10^−1^. Transconjugant counts and conjugation frequencies were determined by culture-based enumeration on selective agar. The recovered transconjugants were verified by MIC testing and endpoint PCR detection of *mcr-1* (Figure S3A,B). Notably, the number of transconjugants and conjugation rate exhibited a dose-dependent reduction following SA treatment (Figure 1A,B). Transconjugant counts were 2.69 × 10^8^, 5.33 × 10^7^, and 4.61 × 10^6^ CFU/mL at 2, 4, and 8 µg/mL SA, respectively. At 8 µg/mL, the conjugation frequency was 1.04 × 10^−3^, corresponding to an approximately 176-fold reduction relative to the control group.

The conjugation of pIncX4-*mcr-1* showed a similar dose-associated reduction. Conjugative frequency decreased by approximately 2-, 7-, and 117-fold at 2, 4, and 8 µg/mL SA, respectively (Figure 1C), and the corresponding transconjugant counts were 1.02 × 10^7^, 2.37 × 10^6^, and 1.43 × 10^5^ CFU/mL (Figure 1D). Collectively, our results demonstrated a substantial and dose-dependent reduction in the conjugation of plasmids carrying *mcr-1* by SA.

### 2.2. SA Limited the Energy Driving Force for Conjugative Transfer

**Figure 2:**
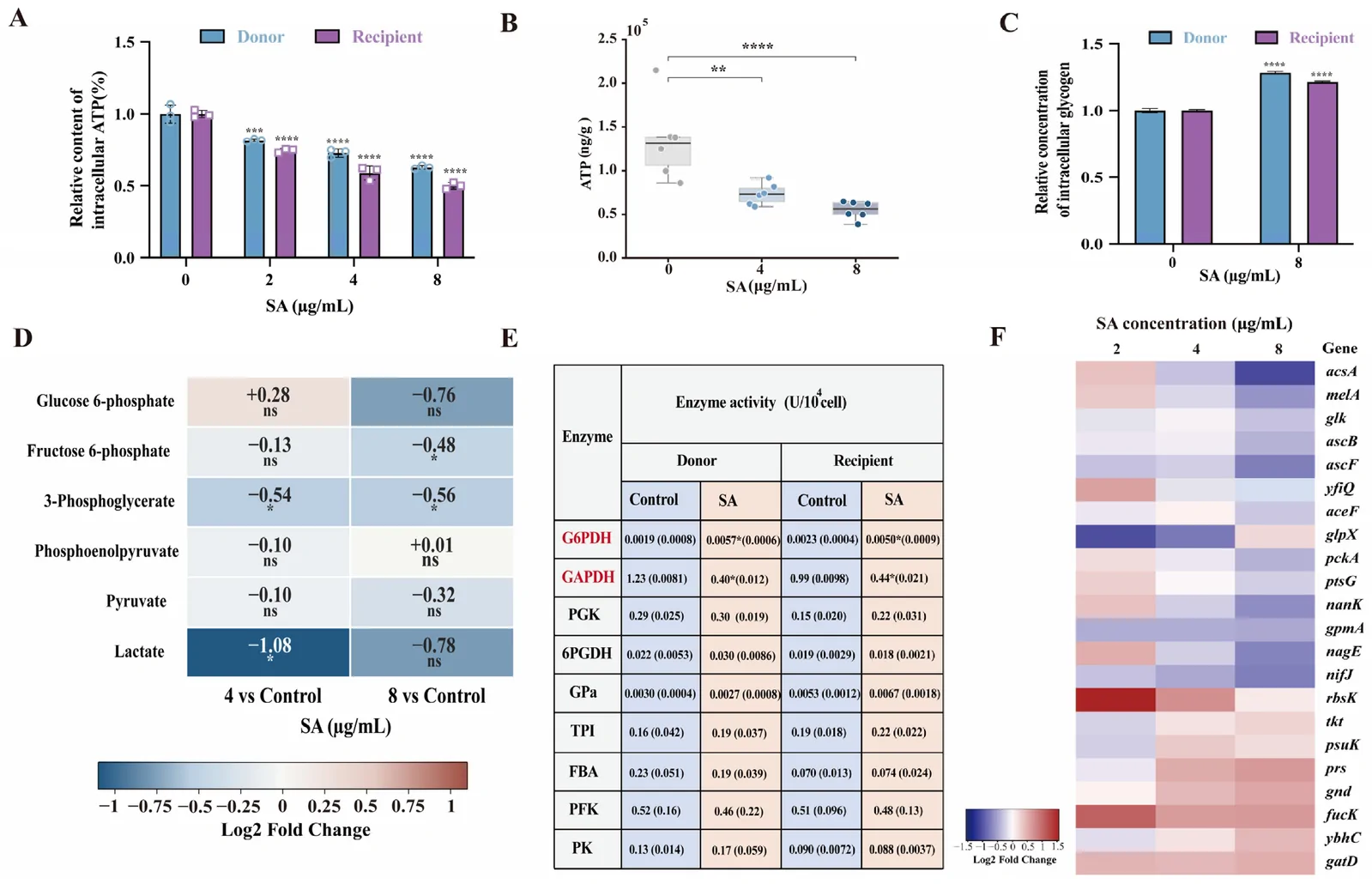
Effect of SA on energy supply for conjugation. (**A**) Relative intracellular ATP signal in donor and recipient bacteria. Circles represent donor strains, whereas squares represent recipient strains. (**B**) Absolute intracellular ATP content determined by metabolomics. (**C**) Relative intracellular glycogen content. (**D**) Abundance of glycolysis-related metabolites. (**E**) Activities of nine assayed enzymes at 8 µg/mL SA, with significant changes observed for GAPDH and G6PDH. (**F**) Selected glycolysis- and pentose-phosphate-pathway-associated transcript changes in donor bacteria. Data are the mean ± standard deviation of at least three biological replicates. Statistical comparisons used one-way ANOVA with Dunnett’s test. Significance levels were defined as follows: ns, not significant, * *p* < 0.05, ** *p* < 0.01, *** *p* < 0.001, and **** *p* < 0.0001.

The luciferase-based ATP signal decreased with increasing SA concentration in donor and recipient cells (Figure 2A). At 4 µg/mL, ATP levels decreased to 73% and 59% of control in donor and recipient strains, and further declined to 63% and 50% at 8 µg/mL. Targeted metabolomics data independently confirmed this reduction (Figure 2B). Mean ATP abundance decreased from 1.34 × 10^5^ ng/g in the control group to 7.35 × 10^4^ and 5.50 × 10^4^ ng/g after treatment with 4 and 8 µg/mL SA, respectively, corresponding to reductions of 45.0% and 58.9%. In addition, an independent arsenate pretreatment, which lowers ATP availability through formation of hydrolysable ADP-arsenate, was also accompanied by a lower conjugation frequency (Figure S4). These results indicate that the reduced conjugation frequency of the *mcr-1*-carrying IncI2 plasmid is closely associated with SA-induced ATP depletion.

At 8 µg/mL SA, glycogen content increased by approximately 28% in donors and 21% in recipients (Figure 2C). This accumulation suggests reduced glycogen catabolism and supports the notion that SA interferes with energy metabolism. In *E. coli*, glycogen is mainly channelled through glycolysis and the pentose phosphate pathway (PPP) (Figure S5A). Of the nine enzyme activities measured, GAPDH activity decreased by 33% in donors and 45% in recipients, whereas G6PDH activity increased approximately 2.9-fold and 2.1-fold, respectively (Figure 2D). The other seven measured enzyme activities did not show marked changes under the tested condition. Targeted metabolomics provided corresponding evidence at the metabolite level (Figure 2E). At 4 µg/mL, 3-phosphoglycerate and lactate decreased by 31.4% and 52.8%, respectively; at 8 µg/mL, fructose 6-phosphate and 3-phosphoglycerate decreased by 28.3% and 32.0%, respectively (Dunnett-adjusted *p* < 0.05). In contrast, phosphoenolpyruvate and pyruvate did not differ significantly from the control, indicating selective depletion rather than a uniform decrease in all measured glycolytic intermediates. Consistent with these enzymatic changes, transcriptomic analysis revealed a coordinated decreases in several glycolysis-associated transcripts and increases in selected pentose-phosphate-pathway-associated transcripts (Figure 2F and Figure S5B, and Tables S1 and S2). The expression pattern was consistent with the enzyme and glycogen findings. Together with the decrease in ATP-associated luminescence, these results suggest that SA may restrict energy availability by suppressing glycolysis, thereby contributing to the reduced conjugative transfer phenotype.

### 2.3. IncI2 Transfer Inhibition Coincided with Lower Transfer-Related Transcript Abundance

**Figure 3:**
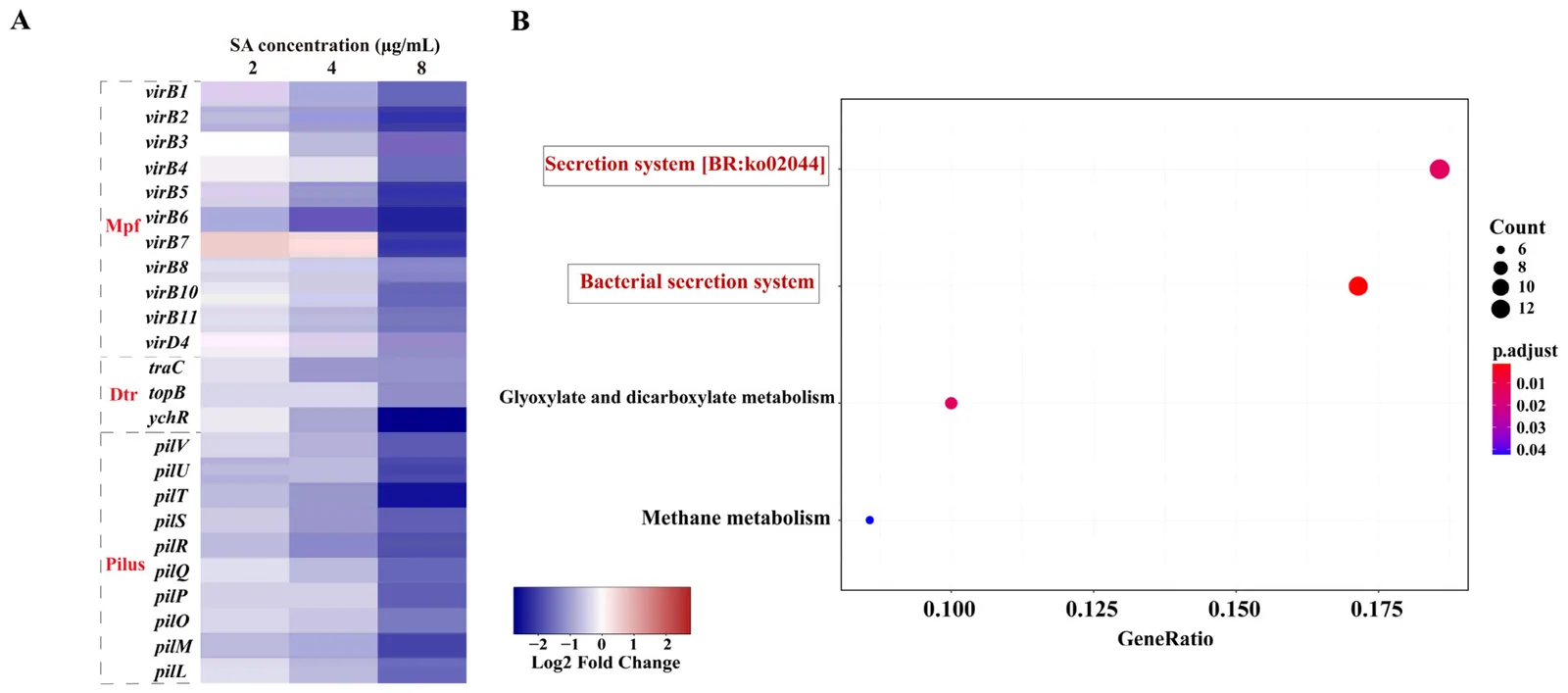
pIncI2-*mcr-1* conjugation-related transcriptional changes associated with SA exposure. (**A**) Relative abundance of selected Dtr-, Mpf-, coupling-, and pilus-associated transcripts. (**B**) KEGG enrichment analysis of downregulated genes at 8 µg/mL SA. Differential-expression criteria were fold change ≥ 2 and FDR < 0.05.

SA exposure was accompanied by concentration-associated decreases in transcripts assigned to the IncI2 DNA-transfer-and-replication (Dtr), mating-pair-formation (Mpf), and coupling functions (Figure 3A and Table S3). The *traC* transcript decreased by 1.2-, 2.1-, and 2.2-fold at 2, 4, and 8 µg/mL SA, respectively, and *yhcR* decreased by 1.2-, 1.9-, and 6.8-fold. Among the Mpf-associated genes, *virB1*, *virB2*, and *virB5* decreased by up to 3.1-, 4.2-, and 4.4-fold, respectively, at 8 µg/mL. The coupling-protein gene *virD4* decreased by 1.1-, 1.4-, and 2.3-fold across the same concentrations. KEGG enrichment analysis of downregulated genes identified enrichment of bacterial secretion system-related pathways (ko03070, 13 counts, q < 0.05) (Figure 3B).

Transcripts associated with the IncI2 type IV pilus also decreased. For example, *pilV* abundance was lower by 1.3-, 1.7-, and 3.3-fold at 2, 4, and 8 µg/mL SA, respectively (Figure 3A and Table S3). Based on these transcriptional changes, it is hypothesized that SA exerts its inhibitory effect on plasmid conjugative transfer by suppressing the expression of pili formation genes and modulating the expression of other regulatory genes involved in conjugation.

### 2.4. Effect of SA on SOS Response and Membrane Permeability

**Figure 4:**
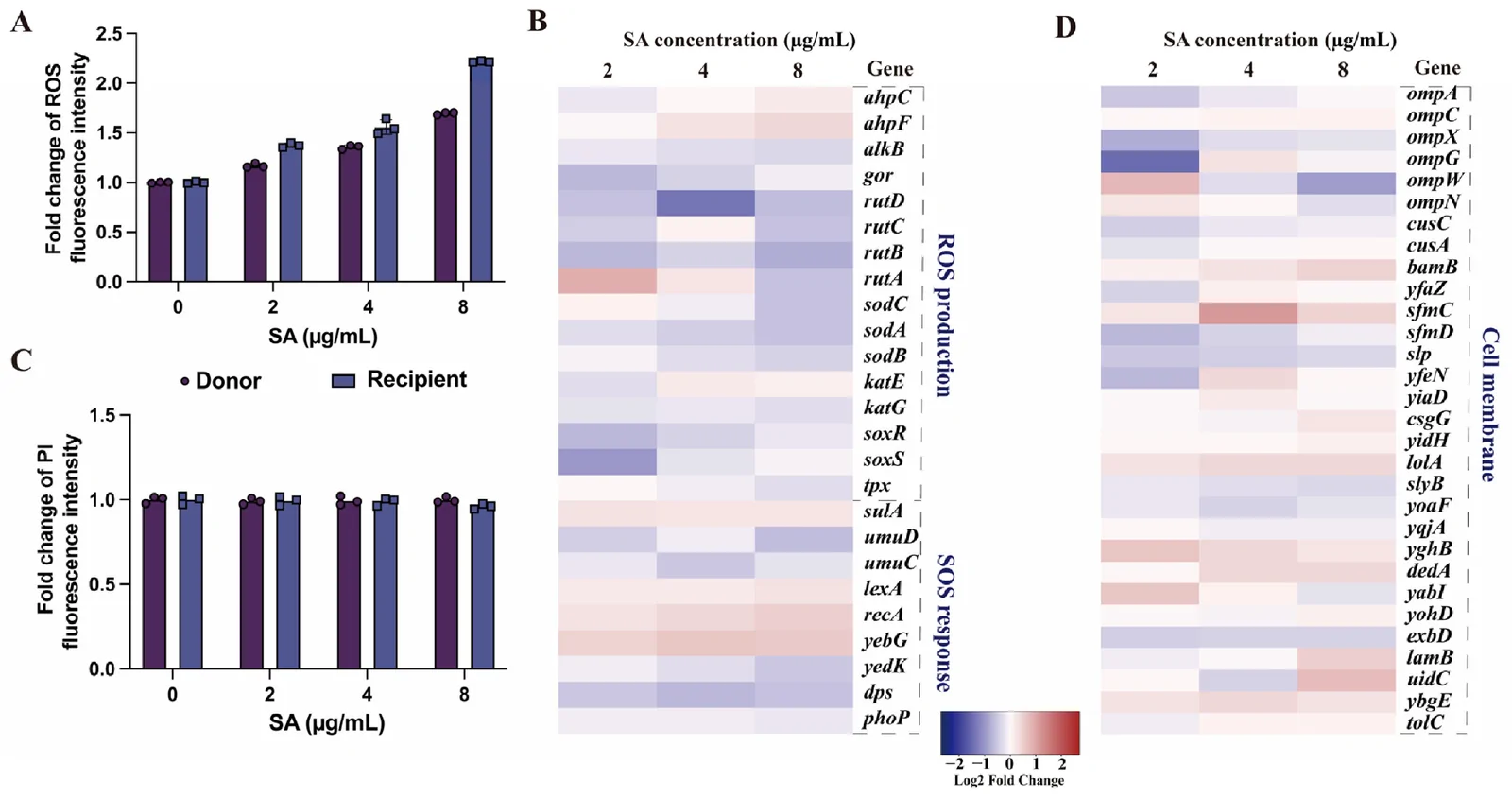
ROS-, SOS-, and membrane-associated measurements after SA exposure. (**A**) Relative ROS-associated fluorescence; (**B**) Selected ROS- and SOS-associated transcripts. (**C**) PI-based membrane-permeability measurement; (**D**) Selected inner- and outer-membrane-associated transcripts. Data are mean ± SD from three biological replicates.

ROS-associated fluorescence increased approximately 1.2- to 2.2-fold in donor and recipient cells across the tested SA concentrations (Figure 4A). However, the selected ROS- and SOS-associated transcripts did not show a coordinated response consistent with strong SOS induction (Figure 4B and Figure S6, and Tables S4 and S5). In contrast to the increase in ROS-associated fluorescence shown in Figure 4A, PI fluorescence did not show a marked increase in either donor or recipient cells, including at the highest SA concentration (Figure 4C). Consistently, the membrane-associated transcripts examined did not show coordinated changes indicative of substantial membrane permeabilization (Figure 4D and Figure S6; Tables S6 and S7). Consequently, we deduced that SA constrained the energy available for conjugation and diminished the expressions of conjugation-related and pilus-related genes, independent of SOS response and the cell membrane.

### 2.5. SA Treatment Was Associated with a Lower IncI2 Transfer-Frequency Estimate in Mice

**Figure 5:**
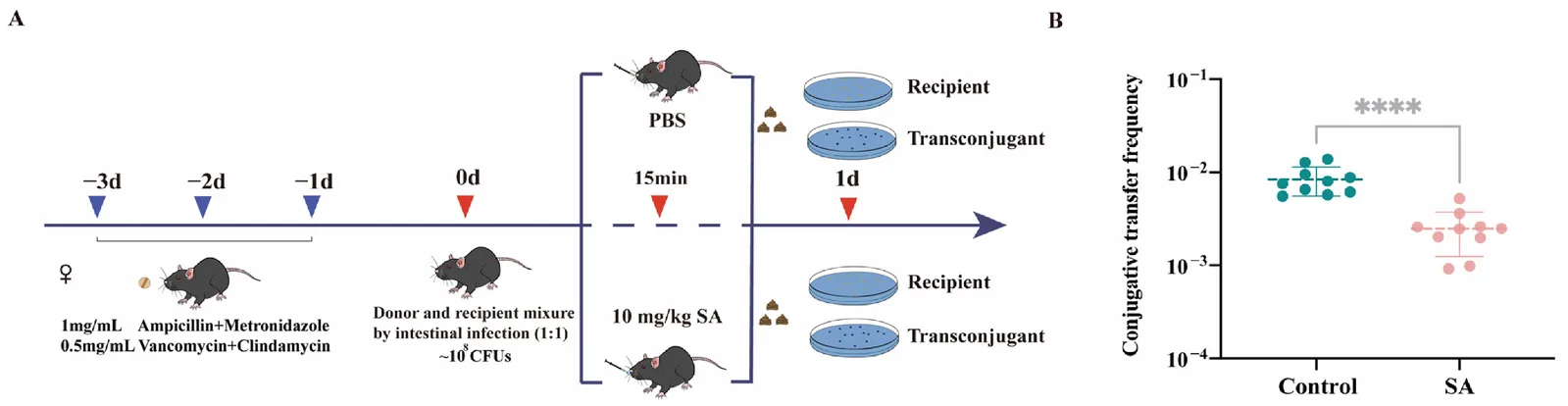
pIncI2-*mcr-1* conjugation-frequency estimates in the mouse-gut assay. (**A**) Scheme of the experimental protocols for conjugation in vivo. (**B**) Calculated conjugation frequency (transconjugants/recipients) in PBS and SA groups (*n* = 10 animals per group). Green and pink circles represent individual animals in the control and SA-treated groups, respectively. The two-sided Mann–Whitney U test was used; **** *p* < 0.0001.

In the mouse-gut assay, the mean IncI2 conjugation-frequency estimate was 8.41 × 10^−3^ in the PBS group and 2.49 × 10^−3^ in the SA group, an approximately threefold difference (*n* = 10 per group; two-sided Mann–Whitney U test, *p* < 0.0001; Figure 5). This result was directionally consistent with the in vitro IncI2 observation. Importantly, donor and recipient colonization levels did not differ significantly between the SA-treated and control groups (Figure S7). These findings indicate that the lower conjugation frequency was not attributable to reduced donor or recipient abundance, supporting an inhibitory effect of SA on IncI2 plasmid transfer in the mouse gut.

## 3. Discussion

This study identifies SA as a candidate inhibitor of conjugative transfer for two epidemiologically important *mcr-1*-carrying plasmids. SA reduced the transfer of both IncI2-*mcr-1* and IncX4-*mcr-1* in a concentration-dependent manner without detectably decreasing bacterial growth or viable donor and recipient counts. At 8 µg/mL, conjugative frequency decreased by approximately 176-fold for pIncI2-*mcr-1* and 117-fold for pIncX4-*mcr-1*. A reduction in pIncI2-*mcr-1* transfer was also observed in the mouse gut, although its magnitude was smaller than that observed in vitro (approximately threefold). Together, these findings establish SA as a promising conjugation inhibitor that can suppress the horizontal dissemination of mobile colistin resistance under both in vitro and intestinal conditions.

The present findings extend the growing evidence that bacterial conjugation can be disrupted by compounds that alter cellular energetics. Dihydroartemisinin, chelerythrine, short-chain fatty acids, and acetylshikonin have been reported to inhibit plasmid transfer through energy-, membrane-, or conjugation-system-associated effects [17,18,19,20]. Our study adds SA to this group and demonstrates substantial activity against two major *mcr-1* plasmid types. IncI2 and IncX4 plasmids contribute extensively to the persistence and dissemination of *mcr-1* across human, animal, food, and environmental reservoirs [7,11,13]. Their response to SA therefore suggests that conjugation inhibition could complement existing strategies aimed at reducing colistin-resistance transmission.

The metabolic results support a mechanism in which SA restricts the energy supply required for conjugative transfer. SA exposure decreased the intracellular ATP-associated signal in both donor and recipient cells, and targeted LC–MS/MS independently confirmed a dose-associated decrease in ATP in the donor. The metabolomics data further showed lower pools of fructose 6-phosphate, 3-phosphoglycerate, and lactate under one or both SA concentrations, whereas phosphoenolpyruvate and pyruvate remained unchanged. This selective pattern indicates that SA does not simply deplete every central-carbon metabolite; instead, it produces a defined redistribution of glycolysis-associated metabolite pools alongside the loss of ATP. In parallel, glycogen accumulated, GAPDH activity decreased, G6PDH activity increased, and the transcriptomic response showed decreased expression of several glycolysis-associated genes with increased expression of selected pentose-phosphate-pathway genes. Taken together, these independent biochemical, metabolomic, enzymatic, and transcriptional measurements support a model in which SA suppresses glycolysis-associated energy production and redirects central carbon metabolism, thereby reducing the ATP available for conjugation. The arsenate experiment reinforces this interpretation because experimentally lowering ATP availability was likewise accompanied by reduced plasmid transfer.

The absence of a detectable growth reduction does not contradict this energy-associated mechanism. Conjugation is an energetically demanding but non-essential process that requires pilus biogenesis, mating-pair stabilization, DNA processing, and substrate transport through the type IV secretion system. A moderate reduction in available cellular energy may therefore affect conjugation before producing an obvious growth defect. Cellular resource allocation is known to influence the balance between bacterial proliferation and horizontal plasmid transmission [24]. Our results suggest that SA shifts this balance away from plasmid transfer while preserving sufficient metabolic capacity for growth under the tested conditions.

SA-induced metabolic changes were accompanied by broad suppression of the IncI2 transfer machinery. Transcripts associated with DNA transfer and replication, mating-pair formation, coupling functions, and type IV pilus formation decreased with increasing SA concentration. These included *traC*, *yhcR*, *virB1*, *virB2*, *virB5*, *virD4*, and *pilV*. KEGG analysis further identified significant enrichment of the bacterial secretion system among the downregulated genes. These observations provide a direct molecular link between the altered energetic state and reduced expression of the apparatus required for conjugation. We therefore propose that SA inhibits IncI2 plasmid transfer through two closely connected processes: restriction of the energy available for conjugation and suppression of genes required for mating-pair formation and plasmid transport. Because plasmid-specific regulators can strongly influence conjugation [25,26], the molecular response of IncX4 should be examined directly in future studies.

The ROS and membrane results help distinguish this mechanism from alternative antimicrobial effects. SA moderately increased ROS-associated fluorescence, but this increase was not accompanied by coordinated activation of ROS- or SOS-response genes. Similarly, neither PI fluorescence nor membrane-associated transcriptional patterns indicated substantial membrane permeabilization. SA therefore appears unlikely to inhibit conjugation primarily through a classical SOS response or gross membrane damage. Instead, the combined metabolic and transcriptomic evidence supports energy restriction and repression of the conjugative machinery as the dominant processes under the tested conditions. The modest ROS increase may reflect a secondary consequence of metabolic redistribution rather than the principal cause of transfer inhibition.

The mouse experiment provided in vivo support for the inhibitory activity of SA, although the magnitude of the effect was substantially smaller than that observed in vitro. SA treatment reduced the mean IncI2 conjugation frequency from 8.41 × 10^−3^ to 2.49 × 10^−3^ in the mouse gut, corresponding to an approximately 3.4-fold reduction, compared with the approximately 176-fold reduction observed at 8 µg/mL in vitro. However, these effect sizes are not directly comparable because of substantial differences in exposure conditions and experimental endpoints. In vitro, donor and recipient cells were uniformly exposed to a defined SA concentration in a controlled mating system. In contrast, oral administration at 10 mg/kg may not produce or maintain an equivalent free SA concentration at the intestinal sites where conjugation occurs because of intestinal absorption, metabolism, degradation, dilution, and binding to luminal contents. In addition, the intestinal microbiota, spatial distribution of donor and recipient cells, and local gradients in nutrients, pH, and oxygen can influence bacterial metabolic states and donor–recipient contact [27,28]. The fecal transfer-frequency estimate may also be affected by bacterial growth, intestinal transit, and clearance. Importantly, donor and recipient abundances did not differ significantly between the SA-treated and control groups, indicating that the lower transfer frequency was not attributable to depletion of either bacterial population. Thus, the in vivo findings support an inhibitory effect in the same direction as that observed in vitro, but not an equivalent magnitude of inhibition. Further studies measuring intestinal SA concentrations and pharmacokinetic profiles are needed to optimize its in vivo anti-conjugation activity.

Several questions remain to be addressed. The current study examined two *mcr-1*-carrying plasmids, and additional plasmid types are needed to define the breadth of SA activity. The proposed metabolic mechanism is supported by ATP, glycogen, enzyme activity, arsenate, and transcriptomic measurements, but direct rescue experiments would further establish causality. Measurements of transfer-protein abundance and pilus assembly would also clarify how transcriptional repression is translated into functional inhibition. In addition, repeated-exposure studies will be important because AcrB-mediated efflux can increase bacterial resistance to SA [23]. The safety and translational relevance of SA also remain to be established. The concentrations used in vitro (2–8 µg/mL) and the oral dose used in mice (10 mg/kg) should not be interpreted as established safe exposures for humans. Previous studies have reported potential cytotoxic and genotoxic effects of SA, and a recent EFSA assessment could not conclude on consumer safety for a Macleaya cordata-derived feed additive [29,30]. Therefore, further pharmacokinetic and systematic toxicological studies are required before considering human application. These priorities define the additional evidence required to develop SA as a practical conjugation-modulating compound.

In conclusion, our findings support a model in which SA suppresses *mcr-1* plasmid dissemination by restricting glycolytic energy production and reducing expression of the conjugative transfer machinery. The strong inhibition of IncI2 and IncX4 transfer in vitro, together with reduced IncI2 transfer in the mouse gut without altered donor or recipient colonization, highlights the potential of SA as an anti-conjugation agent. Targeting plasmid transmission rather than bacterial survival may offer a complementary strategy for limiting the spread of colistin resistance and preserving the clinical utility of this last-line antibiotic.

## 4. Materials and Methods

### 4.1. Study Design

Conjugation frequency was the primary outcome. SA was first tested at 2–8 µg/mL under the reported growth and viable-count conditions, after which conjugation of the pIncI2-*mcr-1* and pIncX4-*mcr-1* were measured. ATP, glycogen, enzyme activity, targeted energy metabolomics, RNA-seq, ROS, and membrane-permeability measurements were used as secondary readouts to characterize biological changes accompanying the pIncI2-*mcr-1* transfer phenotype. These assays used different exposure durations and media, as specified below, and were not treated as a temporal causal sequence. An IncI2 mouse-gut assay provided cross-context evidence for the measured transfer-frequency endpoint.

### 4.2. Sanguinarine, Bacterial Strains, and Plasmids

SA (≥98% purity, Solarbio, Beijing, China) was used in all experiments. The chemical structure of SA is shown in Figure S8. The *mcr-1*-carrying IncI2 plasmid from ZJ807 (BioSample SAMN05437814) and the IncX4-*mcr-1* plasmid from ZJ28 (BioSample SAMN05437813) were introduced into *E. coli* BW25113 by electroporation to generate BW25113/IncI2-*mcr-1* and BW25113/IncX4-*mcr-1* donors. Sodium-azide-resistant *E. coli* J53 served as the recipient in the in vitro assays.

### 4.3. Growth and Viable-Count Measurements

Growth curves were recorded with 0–8 µg/mL SA. Overnight donor and recipient cultures were diluted 1:10 into fresh brain heart infusion (BHI, Luqiao, Beijing, China) broth and incubated at 37 °C for 4 h. Cultures were adjusted to 0.5 McFarland, dispensed into 96-well plates, and incubated with the indicated SA concentrations in an Infinite M200 microplate reader (Tecan, Switzerland) at 37 °C. OD_600_ was recorded every 30 min for 24 h. Donor and recipient viable counts were determined under the reported SA exposures.

### 4.4. In Vitro Conjugation

Donors were grown overnight at 37 °C and 200 rpm in BHI containing 1 µg/mL of colistin (≥98% purity, Solarbio, Beijing, China), and the recipient was grown in BHI containing 100 µg/mL of sodium azide (≥98% purity, Zeye, Shanghai, China). Cultures were diluted 1:10 into 3 mL of fresh broth and incubated for 4 h. Donor and recipient cultures were mixed 1:1 in a final volume of 2 mL, supplemented with SA at the indicated concentration, and incubated without shaking for 16 h at 37 °C. Aliquots (100 µL) from 10-fold serial dilutions were plated on medium containing sodium azide (200 µg/mL) to enumerate recipients and on medium containing colistin (2 µg/mL) plus sodium azide (200 µg/mL) to enumerate transconjugants. Plates were incubated for 24 h at 37 °C. Conjugation frequency was calculated as transconjugants divided by recipients. Transconjugants were checked by susceptibility testing and PCR.

### 4.5. Intracellular ATP Assay

Intracellular ATP-associated luminescence was measured with the Enhanced ATP Assay Kit (Beyotime, Shanghai, China). Overnight cultures were diluted 1:100 into 50 mL of LB broth and incubated at 37 °C for 6 h. Cells were washed, resuspended in 0.1 M phosphate-buffered saline (PBS, pH 7.4) to an OD_600_ of 0.5, and exposed to SA for 1 h at 37 °C. Cells were collected by centrifugation at 15,300 × g for 5 min at 4 °C, resuspended in lysozyme (1 mg/mL, Beyotime, Shanghai, China), and incubated for 15 min at 37 °C. After centrifugation under the same conditions, supernatants were combined with ATP working solution in 96-well plates, incubated for 5 min at 37 °C, and immediately measured with an Infinite M200 reader. This short PBS-based assay differed from the 16 h broth conjugation assay and the 2 h RNA-seq exposure.

### 4.6. Glycogen and Enzyme-Activity Measurements

Intracellular glycogen was quantified using a glycogen assay kit (Solarbio, Beijing, China) according to the manufacturer’s instructions. Activities of nine enzymes related to glycogen utilization and central carbon metabolism were measured: glycogen phosphorylase (Gpa), phosphofructokinase (PFK), fructose-1,6-bisphosphate aldolase (FBA), triosephosphate isomerase (TPI), glyceraldehyde-3-phosphate dehydrogenase (GAPDH), 3-phosphoglycerate kinase (PGK), pyruvate kinase (PK), glucose-6-phosphate dehydrogenase (G6PDH), and 6-phosphogluconate dehydrogenase (6PGDH). Commercial kits were from Solarbio (BC3340, BC0530, BC2270, BC2260, BC2210, BC2250, BC0540, BC0265, and BC2100). Samples exposed to 0 or 8 µg/mL SA were lysed by sonication (200 W, 10 min) on ice. Absorbance at 340 nm was measured after lysate addition to the relevant reaction mixture. Activities were normalized to cell number and expressed as U/10^4^ cells.

### 4.7. Targeted Energy Metabolomics

BW25113/IncI2-*mcr-1* donor cells were exposed to 0, 4, or 8 µg/mL SA on brain heart infusion for 2 h. Bacterial cells were then harvested, and six biological replicates from each group were submitted to Shanghai Applied Protein Technology Co., Ltd. (Shanghai, China) for targeted energy-metabolomics analysis. Metabolites were extracted using pre-cooled methanol/acetonitrile containing isotope-labelled internal standards and analyzed by LC–MS/MS (Shimadzu Corporation, Kyoto, Japan) in multiple-reaction-monitoring mode. Metabolites were identified and quantified using authentic standards, with pooled quality-control samples included throughout the analytical sequence. Metabolite concentrations were normalized to sample mass and expressed as ng/g.

### 4.8. RNA Sequencing and Enrichment Analysis

Total RNA was isolated from conjugation mixtures incubated for 2 h with 0, 2, 4, or 8 µg/mL SA and sequenced using an Illumina HiSeq X Ten platform (Beijing Saimo Lily Biotechnology Co., Ltd., Beijing, China). FASTP (v1.3.7) was used to remove adapter-contaminated reads, reads with >10% ambiguous bases, and reads with >50% of bases at Q < 20. Clean reads were aligned with Bowtie2 (v2.5.5); ribosomal RNA reads were removed; and expression was quantified with RSEM (v1.3.3) as fragments per kilobase of transcript per million mapped reads (FPKM). Differentially expressed genes were identified with edgeR (v4.10.1) using a fold change ≥ 2 and false discovery rate (FDR) < 0.05. Gene Ontology and Kyoto Encyclopedia of Genes and Genomes (KEGG) enrichment analyses used an FDR threshold <0.05.

### 4.9. ROS and Membrane-Permeability Measurements

Intracellular ROS-associated fluorescence was measured with DCFH-DA (Beyotime, Shanghai, China). Overnight cultures were diluted 1:100 into LB broth and incubated at 37 °C for 4–6 h. Cells were harvested by centrifugation at 1700× *g* for 8 min, resuspended in 0.1 M PBS (pH 7.4) to OD_600_ = 0.5, incubated with 10 µM DCFH-DA for 30 min at 37 °C, and washed three times. Aliquots containing 190 µL of labeled cells and 10 µL of SA solution were incubated for 30 min at 37 °C before fluorescence measurement (excitation, 488 nm; emission, 525 nm).

Membrane permeability was assessed with propidium iodide (PI, Beyotime, Shanghai, China). Overnight cultures were diluted 1:100 into LB broth, grown at 37 °C and 200 rpm for 4–6 h, washed twice, and resuspended in 0.1 M PBS to OD_600_ = 0.5. Cells were labeled with 10 µM PI for 30 min at 37 °C and 200 rpm. Aliquots containing 190 µL of labeled cells and 10 µL of SA solution were incubated for 30 min at 37 °C. Fluorescence was measured at excitation/emission wavelengths of 535/615 nm.

### 4.10. Mouse-Gut Conjugation Assay

BALB/c mice aged 8–16 weeks were used. Mice received drinking water containing 1 mg/mL of ampicillin, 0.5 mg/mL of vancomycin, 0.5 mg/mL clindamycin, and 1 mg/mL of metronidazole (Solarbio, Beijing, China) for 3 days. Groups of 10 mice were orally administered approximately 10^8^ CFU of *E. coli* J53, followed 24 h later by an equivalent dose of ZJ807. After both strains had been administered, mice received SA (10 mg/kg) or PBS by daily gavage. Bacterial populations were quantified by plating onto Chinese Blue agar (Luqiao, Beijing, China) containing colistin (2 μg/mL) to select donors, sodium azide (200 μg/mL) to select recipients, or both antibiotics to select transconjugants. Fecal samples were collected daily, homogenized in 1 mL PBS with glass beads, and plated on Chinese Blue agar containing colistin (2 µg/mL), sodium azide (200 µg/mL), or both agents to quantify the selectable populations. Conjugation frequency was calculated as transconjugants divided by recipients.

### 4.11. Statistical Analysis

Data were analyzed with GraphPad Prism (v10.2.3) and are presented as mean ± standard deviation unless otherwise stated. In vitro comparisons were performed using one-way analysis of variance followed by Dunnett’s multiple-comparisons test. Mouse-group comparisons used a two-sided Mann–Whitney U test, with the animal as the experimental unit. Targeted metabolomics used the biological sample as the experimental unit (*n* = 6 per group) and was reanalyzed in Python (v3.14) using SciPy (v1.18.0). The three metabolomics groups were assessed by one-way analysis of variance, followed by two-sided Dunnett-adjusted comparisons of each SA dose with the untreated control. Heatmap values are log2-transformed ratios of group means. Experiments were reported as being performed at least in triplicate, and *p* < 0.05 was considered statistically significant.

## 5. Conclusions

SA markedly inhibited the conjugative transfer of IncI2- and IncX4-type *mcr-1* plasmids in *E. coli* without detectably affecting bacterial growth. Integrated metabolic, enzymatic, and transcriptomic evidence supports a mechanism in which SA suppresses glycolysis, restricts ATP availability, and downregulates the IncI2 conjugative transfer machinery. In mice, SA reduced IncI2 plasmid transfer without altering donor or recipient colonization, confirming its inhibitory activity in the intestinal environment. These findings identify SA as a promising anti-conjugation agent and support targeting plasmid transmission as a complementary strategy to limit the dissemination of colistin resistance.

## Data Availability

The raw sequence data are available in the Genome Sequence Archive of the National Genomics Data Center, China National Center for Bioinformation/Beijing Institute of Genomics, Chinese Academy of Sciences, under accession CRA026761 (https://ngdc.cncb.ac.cn/gsa, accessed on 9 May 2026).

## Supplementary Materials

The following supporting information can be downloaded at https://www.mdpi.com/article/10.3390/antibiotics15100973/s1. Figure S1: Growth curve of donor (A and B) and recipient (C) bacteria under exposure to different of SA; Figure S2: The number of donor (A) and recipient bacteria (B) in responses to different dosages of SA; Figure S3: MIC determination and PCR verification of donor (*E. coli* BW25113/IncI2-*mcr-1* and *E. coli* BW25113/IncX4-*mcr-1*), recipient (*E. coli* J53) and transconjugant under different SA concentrations. (A) The MIC was performed according to EUCAST clinical breakpoints standard. All MIC experiments were conducted with biological triplicates. (B) PCR analysis of plasmid carrying *mcr-1*, followed by the verification of agarose gel electrophoresis; Figure S4: Effect of arsenate pretreatment on conjugation frequency; Figure S5: Proposed routes for the catabolism of glycogen (A) and Fold-change in expression of core cellular EMP-related and PPP-related genes in recipient bacteria (B) under exposure to different of SA; Figure S6: Effect of SA on ROS production, the SOS response and cell membrane permeability. Expression levels of core genes related to ROS production and the SOS response (A), and inner and outer cell membranes (B), in recipient bacteria; Figure S7: The number of donor and recipient bacteria after SA treatment in vivo; Figure S8: Chemical structure of sanguinarine (SA); Table S1: Genes relevant to energy metabolism in donor bacteria after exposure of SA; Table S2: Genes relevant to energy metabolism in recipient bacteria after exposure of SA; Table S3: Genes relevant to conjugation in donor bacteria after exposure of SA; Table S4: Genes relevant to ROS and SOS in donor bacteria after exposure of SA; Table S5: Genes relevant to ROS and SOS in recipient bacteria after exposure of SA; Table S6: Genes relevant to cell membrane in donor bacteria after exposure of SA; Table S7: Genes relevant to cell membrane in recipient bacteria after exposure of SA.

## Abbreviations

The following abbreviations are used in this manuscript:

AMR	antimicrobial resistance
ARG	antibiotic resistance gene
SA	sanguinarine
HGT	horizontal gene transfer
CFU	colony-forming unit
BHI	brain heart infusion
BHA	brain heart infusion agar
MIC	minimum inhibitory concentration
PBS	phosphate-buffered saline
ROS	reactive oxygen species
PI	propidium iodide
PPP	pentose phosphate pathway
Dtr	transfer and replication
Mpf	mating-pair formation
LC–MS/MS	liquid chromatography–tandem mass spectrometry
RNA-seq	RNA-seq
FDR	false discovery rate
KEGG	Kyoto Encyclopedia of Genes and Genomes
