# Supplementary material for: Sanguinarine Reduces Conjugative Transfer of mcr-1-Positive Plasmids in Escherichia coli
Source: Antibiotics (Basel). 2026 Oct 1;15(10):973. doi: 10.3390/antibiotics15100973 (PMC13700708; doi:10.3390/antibiotics15100973)
Supplement: Supplementary file 1 [file antibiotics-15-00973-s001.zip › antibiotics-4559848-supplementary.pdf]

Sanguinarine Reduces Conjugative Transfer of *mcr-1*-positive Plasmids in *Escherichia coli*

Xueyang Wang[1,2], Junfeng Zhang[3], Zhan Lin[1], Penghui Ru[1], Changsong Wang[2,*] and Hewei Zhang[1,*]

1 College of Food and Drugs, Luoyang Polytechnic, Luoyang 471000, China; 202333008@lypt.edu.cn (X.W.); lzhan0410@163.com (Z.L.); penghui_ru@163.com (P.R.)

2 The 989th Hospital of the Joint Logistics Support Force of the Chinese People's Liberation Army, Luoyang 471031, China; 202333008@lypt.edu.cn (X.W.)

3 College of Animal Science and Technology, Henan University of Science and Technology, Luoyang 471000, China; 9906470@haust.edu.cn (J.Z.)

* Correspondence: wangtmmu150@163.com (C.W.); 201818008@lypt.edu.cn (H.Z.)

Supplementary data

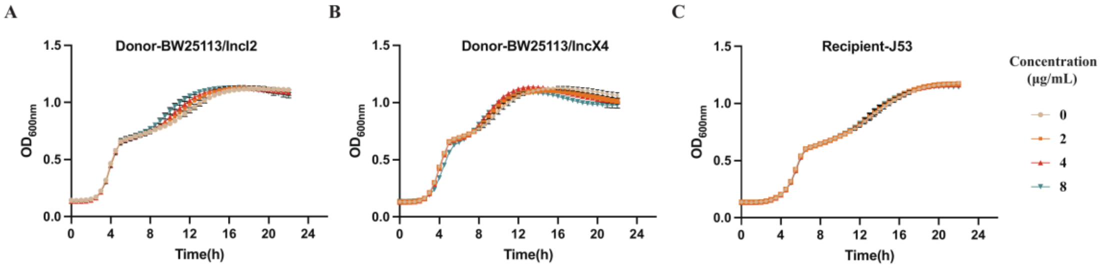

Figure S1. Growth curve of donor (A and B) and recipient (C) bacteria under exposure to different of SA.

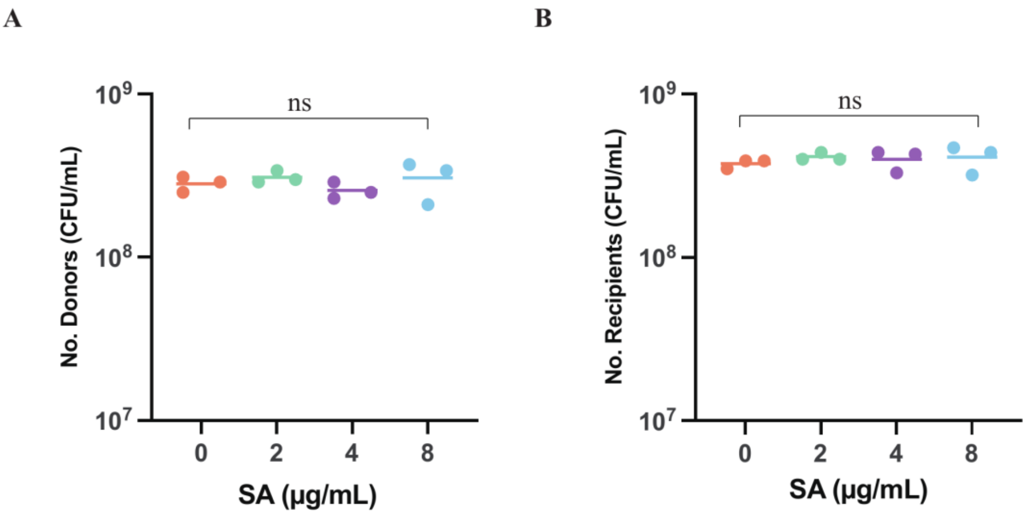

Figure S2. The number of donor (A) and recipient bacteria (B) in responses to different dosages of SA.

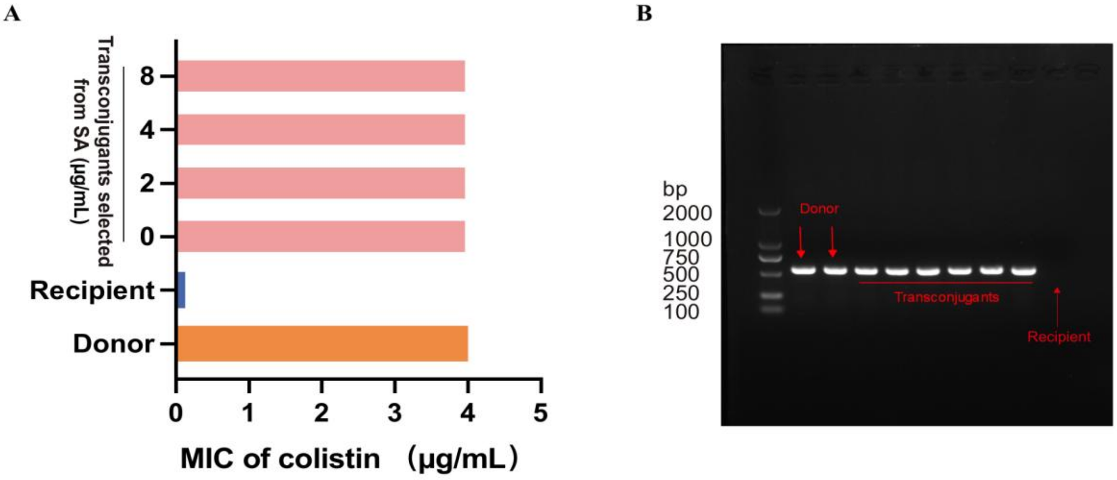

Figure S3. MIC determination and PCR verification of donor (*E. coli* BW25113/IncI2-*mcr-1* and *E. coli* BW25113/IncX4-*mcr-1*), recipient (*E. coli* J53) and transconjugant under different SA concentrations. (A) The MIC was performed according to EUCAST clinical breakpoints standard. All MIC experiments were conducted with biological triplicates. (B) PCR analysis of plasmid carrying *mcr-1*, followed by the verification of agarose gel electrophoresis.

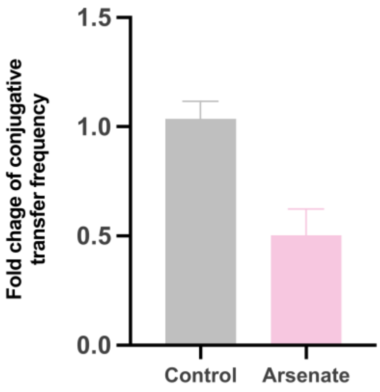

Figure S4. Effect of arsenate pretreatment on conjugation frequency.

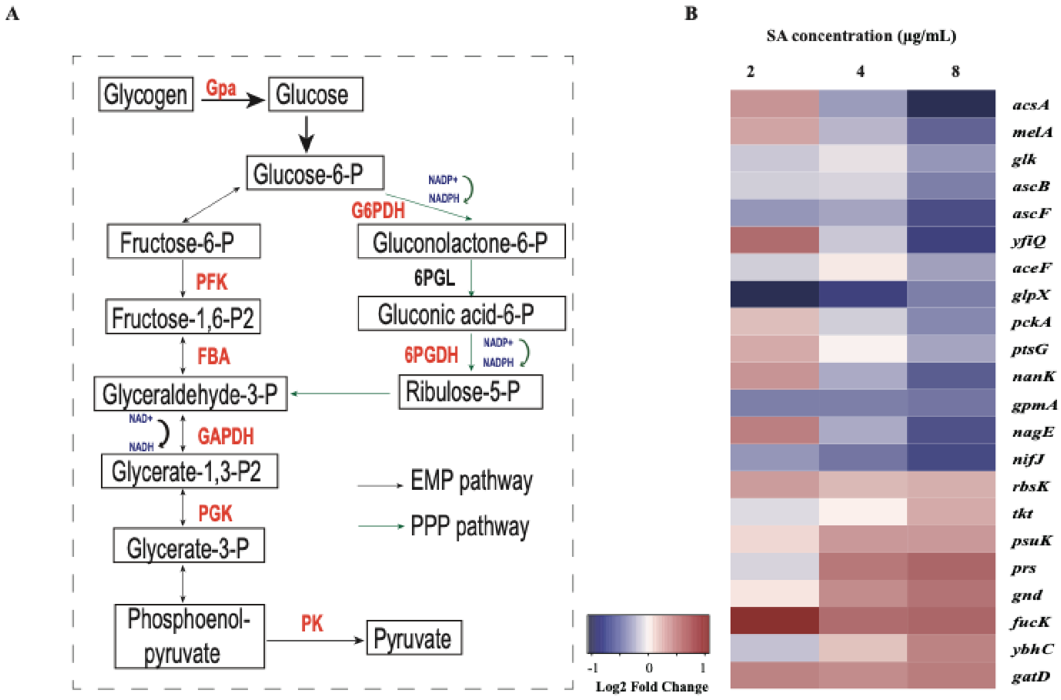

Figure S5. Proposed routes for the catabolism of glycogen (A) and Fold−change in expression of core cellular EMP-related and PPP-related genes in recipient bacteria (B) under exposure to different of SA.

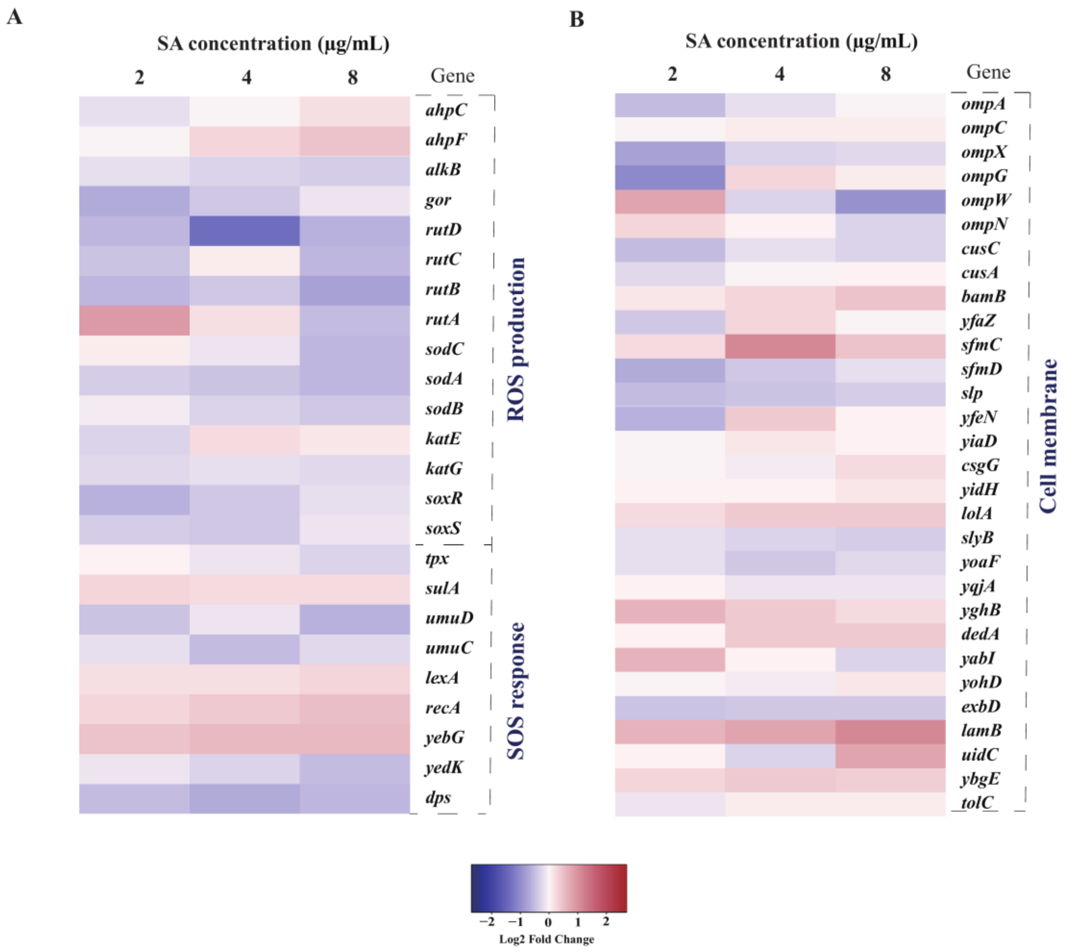

Figure S6. Effect of SA on ROS production, the SOS response and cell membrane permeability. Expression levels of core genes related to ROS production and the SOS response (A), and inner and outer cell membranes (B), in recipient bacteria.

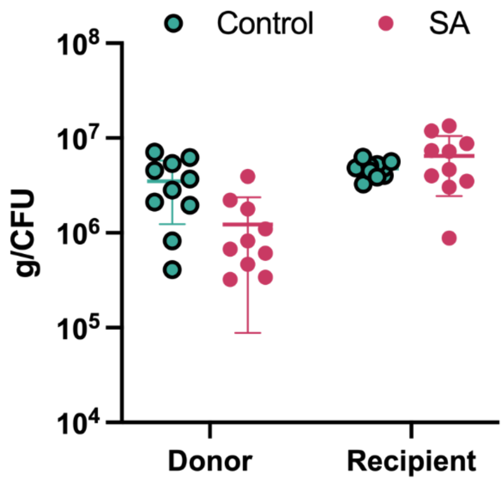

Figure S7. The number of donor and recipient bacteria after SA treatment in vivo.

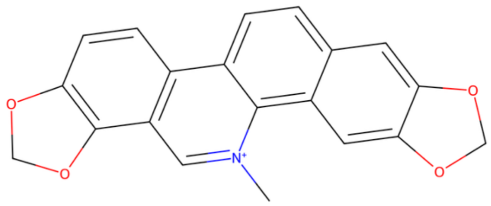

Figure S8 Chemical structure of sanguinarine (SA).

Table S1. Genes relevant to energy metabolism in donor bacteria after exposure of SA.

Gene	Log2(Fold Change of FPKM)		
	**2 μg/mL**	**4 μg/mL**	**8 μg/mL**
acsA	0.38268339	−0.3144972	−1.0637515
melA	0.33082036	−0.2099932	−0.5991432
glk	−0.1470342	−0.0552261	−0.3336315
ascB	−0.1219556	−0.0927856	−0.4309422
ascF	−0.3223545	−0.272153	−0.7263803
yfiQ	0.62093003	−0.1307674	−0.7939019
aceF	−0.1197538	0.04110401	−0.3052977
glpX	−1.0308246	−0.7715753	0.23308092
pckA	0.20203466	−0.1216563	−0.4127502
ptsG	0.29728149	−0.0106584	−0.2718907
nanK	0.39645689	−0.2512057	−0.6238738
gpmA	−0.4385725	−0.4363679	−0.486357
nagE	0.51127262	−0.253792	−0.7084712
nifJ	−0.3370045	−0.4923585	−0.7431597
rbsK	1.55714386	0.72014184	0.10140685
tkt	−0.2279313	0.18463197	0.27805514
psuK	−0.2684383	0.31212397	0.19156687
prs	−0.0968902	0.5563299	0.67616346
gnd	0.05339676	0.44148844	0.58314218
fucK	1.07094682	0.62906916	0.65997055
ybhC	−0.1683709	0.17807098	0.46343224
gatD	0.47932917	0.43761133	0.50600412

Table S2. Genes relevant to energy metabolism in recipient bacteria after exposure of SA.

Gene	Log2(Fold Change of FPKM)		
	**2 μg/mL**	**4 μg/mL**	**8 μg/mL**
acsA	0.38907616	−0.3150952	−1.0295181
melA	0.31994858	−0.2110754	−0.5883182
glk	−0.1443998	−0.0580702	−0.328565
ascB	−0.1281368	−0.1035368	−0.4362644
ascF	−0.3401931	−0.2763085	−0.7202602
yfiQ	0.62232781	−0.1324061	−0.7876809
aceF	−0.1185808	0.03790419	−0.2968192
glpX	−1.0314566	−0.7758408	−0.434066
pckA	0.20179887	−0.1223277	−0.4054194
ptsG	0.2968369	−0.0162828	−0.2678435
nanK	0.39484362	−0.2417921	−0.6234068
gpmA	−0.436044	−0.4399707	−0.4808209
nagE	0.51287188	−0.2565944	−0.7049582
nifJ	−0.3350174	−0.4919523	−0.7304987
rbsK	0.34999817	0.22811981	0.2749619

tkt	−0.080277	0.01086469	0.28266242
psuK	0.09021057	0.3675683	0.38475682
prs	−0.0906917	0.55129876	0.68256876
gnd	0.05339961	0.43439813	0.58485458
fucK	1.07180641	0.63770498	0.67374291
ybhC	−0.1637243	0.18581391	0.4766623
gatD	0.4809906	0.43072629	0.5167167

Table S3. Genes relevant to conjugation in donor bacteria after exposure of SA.

Gene	Log2(Fold Change of FPKM)		
	2 μg/mL	4 μg/mL	8 μg/mL
virB1	−0.4872681	−0.8683762	−1.6483979
virB2	−0.8278303	−1.0337788	−2.0726765
virB3	−0.0055567	−0.8251535	−1.5282569
virB4	−0.1641097	−0.3245632	−1.5868984
virB5	−0.4691499	−1.1199915	−2.1492491
virB6	−0.8447334	−1.6744048	−2.3566859
virB8	0.56943641	0.39580925	−2.0955164
virB9	−0.4146152	−0.5203194	−1.2943924
virB10	−0.2584005	−0.5440717	−1.6452961
virB11	−0.3399734	−0.772461	−1.4472759
virD4	−0.1567268	−0.4829351	−1.1724708
traC	−0.3149258	−1.0587015	−1.110765
topB	−0.4057049	−0.4068799	−1.2013833
yhcR	−0.2066994	−0.9346913	−2.7643536
PilV	−0.3987898	−0.8047773	−1.7231933
PilU	−0.8292858	−0.7105403	−1.8818978
PilT	−0.7229918	−1.1554346	−2.53796
PilS	−0.4977933	−1.0715195	−1.6733453
pilR	−0.6694381	−1.2473731	−1.8574385
PilQ	−0.3578329	−0.6943249	−1.5755391
PilP	−0.4442962	−0.480501	−1.6679552
PilO	−0.395772	−0.5691304	−1.4119095
PilM	−0.8002217	−0.9584546	−1.9023622
pilL	−0.346773	−0.740753	−1.5888358

Table S4. Genes relevant to ROS and SOS in donor bacteria after exposure of SA.

Gene	Log2(Fold Change of FPKM)		
	**2 μg/mL**	**4 μg/mL**	**8 μg/mL**
ahpC	−0.1737885	0.00073734	0.19914365
ahpF	−0.0158421	0.29846276	0.43014598
alkB	−0.167901	−0.2735331	−0.3490509
gor	−0.6860141	−0.3919502	−0.1258136
rutD	−0.5922883	−1.3666143	−0.6225353
rutC	−0.4434872	0.09128297	−0.591262
rutB	−0.6539523	−0.4024154	−0.7696567
rutA	0.90071119	0.2400257	−0.5441739
sodC	0.06174537	−0.129742	−0.5687278
sodA	−0.3177564	−0.4397146	−0.572181
sodB	−0.065273	−0.3008585	−0.4080996
katE	−0.2861724	0.21524524	0.11144957
katG	−0.2182888	−0.2036906	−0.2768925
soxR	−0.6551748	−0.3888086	−0.2159142
soxS	−1.004637	−0.23359	−0.1019028
tpx	0.03467774	−0.1330924	−0.322227
sulA	0.31301806	0.24433852	0.23739286
umuD	−0.4465202	−0.1529522	−0.6385383
umuC	−0.1837103	−0.53375	−0.2636744
lexA	0.19874507	0.21078893	0.29759049
recA	0.28034441	0.4143964	0.50841453
yebG	0.47844462	0.59419957	0.57466479
yedK	−0.1343384	−0.3073624	−0.4932257
dps	−0.5016279	−0.6599298	−0.578085
phoP	−0.1507184	−0.1225046	−0.207617

Table S5. Genes relevant to ROS and SOS in recipient bacteria after exposure of SA.

Gene	Log2(Fold Change of FPKM)		
	**2 μg/mL**	**4 μg/mL**	**8 μg/mL**
ahpC	−0.1744863	−0.0047919	0.20260087
ahpF	−0.0144931	0.29539403	0.43584381
alkB	−0.1631871	−0.3012269	−0.3604637
gor	−0.6832062	−0.3952272	−0.1160005
rutD	−0.5929281	−1.3716064	−0.6190498
rutC	−0.4400445	0.09054784	−0.5857859
rutB	−0.5921574	−0.4046277	−0.7626392
rutA	0.90240031	0.19239643	−0.5386435
sodC	0.07362288	−0.1287226	−0.5619719
sodA	−0.3244946	−0.4511105	−0.5675223
sodB	−0.0624196	−0.3029997	−0.4014011
katE	−0.2776644	0.22119716	0.12230222

katG	−0.2163708	−0.2072856	−0.2691914
soxR	−0.6444122	−0.3917694	−0.202027
fsoxS	−0.3320644	−0.4137472	−0.1413224
tpx	0.03773315	−0.1368943	−0.3130369
sulA	0.31295422	0.24180028	0.23978518
umuD	−0.444758	−0.1344244	−0.6128061
umuC	−0.1711279	−0.5359837	−0.2518846
lexA	0.19633438	0.20179879	0.28997036
recA	0.28502725	0.41650376	0.51773869
yebG	0.48517776	0.58768157	0.58402581
yedK	−0.1574428	−0.3044467	−0.521159
dps	−0.4998834	−0.6628021	−0.5728352
phoP	−0.1744863	−0.0047919	0.20260087

Table S6. Genes relevant to cell membrane in donor bacteria after exposure of SA.

**Gene**	**Log2(Fold Change of FPKM)**		
	**2 μg/mL**	**4 μg/mL**	**8 μg/mL**
ompA	−0.5115869	−0.1845316	−0.0250452
ompC	0.00328004	0.08864587	0.07309592
ompX	−0.8020848	−0.2950441	−0.2549755
ompG	−1.4776573	0.2940608	−0.1036053
ompW	0.78946844	−0.3050347	−0.9388112
ompN	0.24162142	0.05266416	−0.2967983
cusC	−0.4614434	−0.1816375	−0.1579292
cusA	−0.2248458	−0.0218315	0.02171181
bamB	0.1444368	0.30241661	0.44626916
yfaZ	−0.411906	0.11656746	−0.0301756
sfmC	0.26024992	1.13755541	0.43563423
sfmD	−0.6849867	−0.3957207	−0.1459451
slp	−0.5038943	−0.4738496	−0.3578587
yfeN	−0.6924097	0.40035077	0.00733775
yiaD	−0.052274	0.17252837	−0.0101114
csgG	−0.0135899	−0.0661885	0.24032275
yidH	0.04179161	0.01693475	0.12960199
lolA	0.27176709	0.41280158	0.37866977
slyB	−0.1679526	−0.2742564	−0.3545385
yoaF	−0.2057902	−0.4220522	−0.246616
yqjA	0.0026325	−0.1129384	−0.1173762
yghB	0.60319075	0.42204287	0.2374676
dedA	0.05195296	0.40794574	0.37887334
yabI	0.62501981	0.06896753	−0.2686387
yohD	−0.0261171	−0.063285	0.10997322
exbD	−0.4335706	−0.4231231	−0.4240942
lamB	−0.1311594	−0.0422931	0.50526684

uidC	0.01021799	−0.4161213	0.71774776
ybgE	0.2801181	0.38459507	0.31737425
tolC	−0.1526189	0.08159784	0.06246077

Table S7. Genes relevant to cell membrane in recipient bacteria after exposure of SA.

Gene	Log2(Fold Change of FPKM)		
	2 μg/mL	4 μg/mL	8 μg/mL
ompA	−0.5113692	−0.1879929	−0.0200474
ompC	−0.0004418	0.0849513	0.07856777
ompX	−0.800328	−0.2980302	−0.2493731
ompG	−1.0275744	0.29255804	0.09875775
ompW	0.79216934	−0.3128942	−0.9249406
ompN	0.28378669	0.01382914	−0.2911566
cusC	−0.5230697	−0.1846201	−0.270828
cusA	−0.2170131	−0.0319405	0.02753216
bamB	0.14468957	0.2991833	0.44859163
yfaZ	−0.4124541	0.30702196	−0.0262151
sfmC	0.26039714	1.13161735	0.43800563
sfmD	−0.6848018	−0.3989516	−0.1641196
slp	−0.5018747	−0.4764546	−0.3515734
yfeN	−0.6102608	0.39664214	0.01446629
yiaD	−0.0250621	0.16154876	0.00851467
csgG	−0.0127576	−0.0889129	0.24691762
yidH	0.04654034	0.02767899	0.14852324
lolA	0.26869938	0.40855738	0.38310117
slyB	−0.1671961	−0.2761333	−0.3468245
yoaF	−0.2052184	−0.4197915	−0.2272997
yqjA	0.00597592	−0.1145798	−0.1133037
yghB	0.60515848	0.42085585	0.242723
dedA	0.05100401	0.39929786	0.38021921
yabI	0.62119095	0.02556701	−0.2827121
yohD	−0.0230908	−0.0609125	0.12600419
exbD	−0.4378216	−0.4288169	−0.4189796
lamB	0.63278447	0.79246366	1.11852358
uidC	0.00285711	−0.3080904	0.76474764
ybgE	0.28048654	0.38108715	0.33035352
tolC	−0.1487153	0.07769745	0.06886503
